# Effects of PI3Kγ overexpression in the hippocampus on synaptic plasticity and spatial learning

**DOI:** 10.1186/s13041-014-0078-6

**Published:** 2014-11-06

**Authors:** Jun-Hyeok Choi, Pojeong Park, Gi-Chul Baek, Su-Eon Sim, SukJae Joshua Kang, Yeseul Lee, Seo-Hee Ahn, Chae-Seok Lim, Yong-Seok Lee, Graham L Collingridge, Bong-Kiun Kaang

**Affiliations:** Department of Biological Sciences, College of Natural Sciences, Seoul National University, Bldg 504 Rm 202, 599 Gwanangno, Gwanak-gu, Seoul 151-747 Korea; Department of Brain and Cognitive Sciences, College of Natural Sciences, Seoul National University, Seoul, Korea; Department of Life Science, Chung-Ang University, Seoul, Korea; Centre for Synaptic Plasticity, School of Physiology and Pharmacology, University of Bristol, Bristol, BS8 1TD UK

## Abstract

Previous studies have shown that a family of phosphoinositide 3-kinases (PI3Ks) plays pivotal roles in the brain; in particular, we previously reported that knockout of the γ isoform of PI3K (PI3Kγ) in mice impaired synaptic plasticity and reduced behavioral flexibility. To further examine the role of PI3Kγ in synaptic plasticity and hippocampus-dependent behavioral tasks we overexpressed p110γ, the catalytic subunit of PI3Kγ, in the hippocampal CA1 region. We found that the overexpression of p110γ impairs NMDA receptor-dependent long-term depression (LTD) and hippocampus-dependent spatial learning in the Morris water maze (MWM) task. In contrast, long-term potentiation (LTP) and contextual fear memory were not affected by p110γ overexpression. These results, together with the previous knockout study, suggest that a critical level of PI3Kγ in the hippocampus is required for successful induction of LTD and normal learning.

## Introduction

Phosphoinositide 3-kinases (PI3Ks) are involved in various biological processes such as growth, proliferation and differentiation of cells [1,2]. PI3Ks produce various phosphorylated phosphoinositides, including PtdIns3P, PtdIns(3,4)P2, PtdIns(3,5)P2, and PtdIns(3,4,5)P3; all of which function in membrane trafficking and signal transduction [3,4]. The PI3K family is grouped into three classes, I, II and III, according to their structures and substrates. The class I PI3Ks are further divided into IA and IB classes based on the similarities of their sequences. PI3Kγ, the sole member of class IB, is a heterodimer of the p110γ catalytic subunit and either p101 or p84 regulatory subunits, which are encoded by separate genes [1]. Unlike other types of PI3Ks, which have a widespread tissue distribution, PI3Kγ is exclusively expressed in the immune system, the cardiovascular system, and the central nervous system [2]. The roles of PI3Kγ have primarily been studied in the field of immunology where it was first identified in the study of polyoma viruses [3].

Although mounting evidence has revealed that the members of PI3K family are required for learning and memory [4-8], the isoform-specific roles of PI3Ks are still largely undetermined. Recently, we found that PI3Kγ knockout mice (*pi3kcg*^*−/−*^) had a selective impairment in NMDA receptor (NMDAR)-dependent LTD and reversal learning in the Morris water maze (MWM) task [9]. To investigate the function of PI3Kγ in more detail, we have now used a gain-of-function approach by overexpressing PI3Kγ and have studied hippocampus-dependent learning and synaptic plasticity. We found that NMDAR-LTD, but not NMDAR-LTP, was impaired in slices prepared from mice in which PI3Kγ had been overexpressed in the hippocampus. Moreover, performance in a spatial learning task was reduced by PI3Kγ overexpression, while contextual fear memory was not affected. Our findings further support a role of PI3Kγ in some, but not all, forms of hippocampus-dependent synaptic plasticity and spatial learning.

## Materials and methods

### AAV production and stereotactic viral injection

All adeno-associated viruses (AAVs) were produced with AAV2/1 serotyped vectors and packaged using HEK293T cells. Viral titers were determined by quantitative real-time PCR (Prism 7300, Applied Biosystems, USA) with intercalater SYBR green (TAKARA, Japan).

All surgical procedures were conducted in sterile conditions and approved by the Institutional Animal Care and Use Committees of Seoul National University. Wild-type C57BL/6 male mice (8 weeks of age) were anesthetized by an intraperitoneal injection of ketamine and arranged in a stereotactic frame (Stoelting Co. USA). The hippocampal CA1 region (AP: −1.8 mm, ML: ±1.5 mm, DV: −1.7 mm) was targeted, and equivalent amounts of AAVs (3.5 × 10^9^ particles in 1 μl for flag-p110γ and tdTomato) were delivered bilaterally using a 10-μl syringe pump (30 gauge, Hamilton Co., USA) at a rate of 6.0 μl/h. After an additional 10 min of diffusion time, the needle was withdrawn and the scalp was sutured with black silk. AAV vectors were then expressed for 2 weeks.

### Histology, immunohistochemistry and fluorescence imaging

The mice were kept under isoflurane anesthesia and transcardially perfused with pre-chilled 4% paraformaldehyde (PFA) in PBS. Brains were fixed again in 4% PFA at 4°C overnight and then dehydrated in 30% sucrose-PBS solution at 4°C for 2 days. Coronal brain slices were prepared (30 μm for flag-p110γ, 50 μm for tdTomato) by cryosectioning (Leica Ltd., Germany), 50-μm sections for tdTomato were mounted on a slide glass with Vectashield containing DAPI (Vector Lab, USA), and 30-μm sections for flag-p110γ were stored in 50% glycerol in PBS at −20°C.

Flag-p110γ was detected with anti-Flag antibody (Sigma, USA, Cat# F7425, 1:1,000) and goat anti-rabbit Alexa Fluor 488 IgG (Invitrogen, USA, Cat# A11008, 1:500). Fluorescent mages were acquired using an IX51 inverted fluorescent microscope (Olympus, Japan).

### Electrophysiology

#### Hippocampal slice preparation

Transverse hippocampal slices (400 μm) were prepared from C57BL/6 mice aged 12 weeks (24 days after AAV infusion). The animals were anaesthetized with isoflurane and decapitated for the removal of the brain. Brains were then placed in ice-chilled artificial cerebrospinal fluid (ACSF, 124 mM NaCl, 3 mM KCl, 26 mM NaHCO_3_, 1.25 mM NaH_2_PO_4_, 10 mM MgSO_4_, 10 mM Glucose, 1 mM CaCl_2_, aerated with 95% O_2_ and 5% CO_2_). The hippocampus was removed from the brain while maintained in the cold ice solution and sliced using a VT1000 slicer (Leica Ltd., Germany). Hippocampal slices were transferred to ACSF containing 2 mM MgSO_4_ and 2 mM CaCl_2_, where they were allowed to recover at 32°C for 30 min and then maintained at 28°C before recordings were conducted.

#### Slice electrophysiology

Extracellular recordings were performed in an interface chamber (Campden Instrument Ltd., UK) maintained at 32°C. ACSF (124 mM NaCl, 3 mM KCl, 26 mM NaHCO_3_, 1.25 mM NaH_2_PO_4_, 2 mM MgSO_4_, 10 mM glucose, 2 mM CaCl_2_, aerated as above) was perfused continuously at a rate of 2 ml/min. Standard extracellular recordings were performed in the CA1 region of the hippocampal slices to measure the slope of evoked field excitatory postsynaptic potentials (fEPSPs). Responses were obtained using an Axopatch 200B amplifier (Molecular Devices, US) and digitized with a Digidata 1322A A/D board at a sampling rate of 10 kHz. Recordings were monitored and analyzed using WinLTP (WinLTP Ltd. (winltp.com), and The University of Bristol, UK).

#### Synaptic plasticity

A bipolar stimulating electrode was placed in the stratum radiatum of the CA1 region. Baseline responses at a frequency of 0.033 Hz were obtained using a stimulus intensity (between 5 to 20 μA; 0.1 ms pulse width) that evoked responses of approximately 40% of the maximum fEPSP value. Following a stable baseline period of at least 20 min, LTP and LTD were investigated by delivering high-frequency stimulation (100 Hz, 1 s) and low-frequency stimulation (1 Hz, 15 min), respectively. In terms of depotentiation experiments, LTP was induced using three trains of theta burst stimulations (consisting of five pulses at 100 Hz and repeated five times at 5 Hz), with a 10 s inter-train interval. After 30 min of LTP induction, low-frequency stimulation (2 Hz, 10 min) was given to depotentiate the responses.

#### Statistical analysis

All experimental procedures were performed in a blind fashion. The data are presented as the mean ± SEM, and statistical significance was determined using Student’s t-test, and the level of significance was set at *p <0.05 and **p <0.01. To compare the level of synaptic plasticity between the groups, the average of fEPSP responses obtained from the last 5 min period was used.

### Western immunoblotting

For western immunoblotting, we injected flag-p110γ or tdTomato expressing AAV, together with GFP expressing AAV in the CA1 region of 3 weeks-old C57BL/6 male mice. 2 weeks after injection, CA1 regions expressing green fluorescence were dissected and recovered in ACSF maintained at 32°C for 2 h. CA1 mini slices were treated with NMDA (20 μM) for 3 min to induce chemical LTP and immediately snap-frozen in liquid nitrogen. Slices were homogenized using a dounce homogenizer with lysis buffer (10 mM Tris–HCl, 150 mM NaCl, 1 mM EDTA, 1% NP-40, 0.1% SDS, pH 7.5) containing both protease inhibitor cocktail (Roche) and phosphatase inhibitor cocktail (Roche). Samples were analyzed by western blotting using the following antibodies: anti-phospho-p38 MAPK (Cell Signaling) and anit-p38 MAPK (Cell Signaling).

### Behavioral experiment

Male C57BL/6 mice were housed two or four per cage and maintained on a 12/12 h light–dark cycle. Food and water were provided *ad libitum*.

#### Open field test

The mice were exposed to the open field box, a square opaque white box (40 × 40 × 40 cm), for 10 min under dim light. Locomotor activity was monitored and analyzed with EthoVision 3.1 (Noldus, Netherlands).

#### Elevated plus maze task

The elevated plus maze was made of white Plexiglas and consisted of two opposing open arms and two closed arms. All arms were 150 × 5 cm (length × width) and closed arms were enclosed with 20-cm walls. The maze was placed 30 cm above the floor. Mice were placed in the center of the maze and tracked for 5 min by EthoVision 3.1 (Noldus, Netherlands).

#### Morris Water Maze (MWM) task

The water maze was a circular opaque grey tank, 140 cm in diameter, 100 cm in height, filled with water containing small amount of white paint to hide the platform (22 ~ 23°C) to a depth of 30 cm. The maze was surrounded by four distinct visual cues, and all procedures were performed under dim light. A circular, 10 cm in diameter, Perspex platform was positioned in the middle of the virtual quadrant (distance of 35 cm from the edge and 35 cm to the center of the maze) and submerged approximately 1.5 cm below the surface of the water. Each training session consisted of four 60-s trials with four different starting points that were randomly selected and equally distributed among the four quadrants. The mice underwent one training session per day. When the mice reached the platform, they were allowed to remain on the platform for 20 s and then transferred to a holding-cage for 60 s. If a mouse failed to locate the platform within 60 s, it was manually guided to the platform. For the probe test, on day 6, the mice were allowed 60 s of free-swimming without the platform. All experiments were recorded and tracked using Ethovision 3.1 (Noldus, Netherlands).

#### Contextual fear conditioning

Contextual fear memory was measured in a standard chamber (Freeze Frame, Coulbourn) after 4 days of handling. The mice were placed in the fear chamber for 5 min. After 2 min, the mice were given three consecutive foot shocks (2 s, 0.24 mA, 1 min intervals). After 24 h, the mice were again placed in the same chamber for memory retrieval.

## Results

### Electrophysiological properties of p110γ overexpressed hippocampal slices

**Figure 1:**
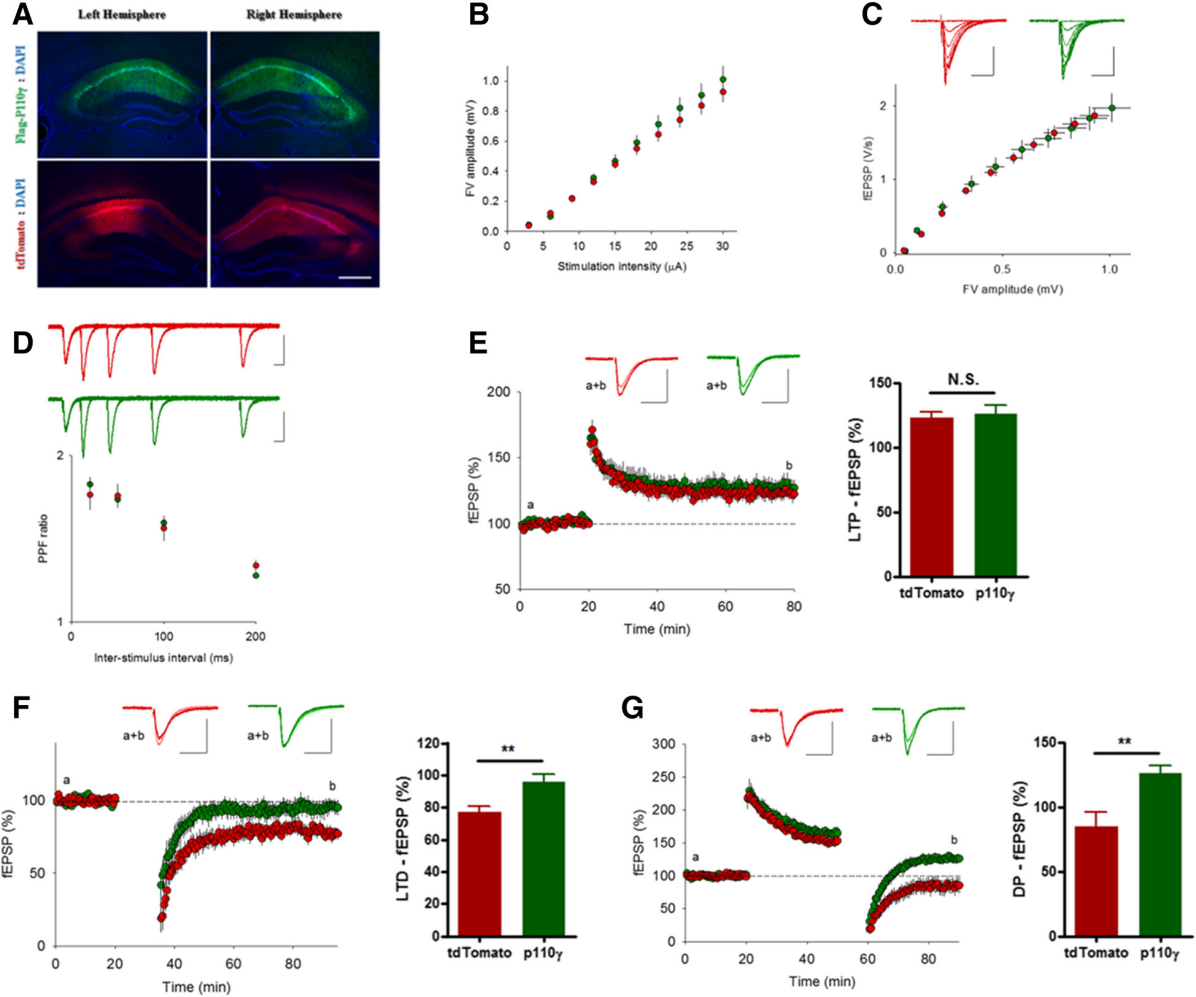
**NMDAR-LTD and depotentiation are inhibited by p110γ Overexpression in the hippocampal CA1 Area. (A)** Representative images of Flag-tagged p110γ or tdTomato expression in the mouse hippocampal CA1 area. (Blue: DAPI, Green: Flag-p110γ, Red: tdTomato, imaged 50 days after AAV injection). Scale bar represents 500 μm. **(B, C)** Input/output relationship was unaltered in p110γ-overexpressed mice (green symbols) compared to control group (tdTomato; red symbols). The stimulation intensity was varied by 3 μA increments, and the insets indicate representative traces (averages of 4 successive sweeps; scale bars: 0.5 mV, 10 ms in this and the following panels). **(D)** Paired-pulse facilitation (PPF) in p110γ mice was unaffected; the measured inter-stimulus intervals were 20, 50, 100 and 200 ms. **(E)** Excessive p110γ had no effect on LTP induced by a brief tetanus (100 Hz, 1 s). Typical traces are obtained at the times indicated by a (light trace) and b (thick trace). **(F)** NMDAR-LTD induced by LFS (1 Hz, 15 min) was impaired in p110γ mice (p <0.01, Student’s t-test). **(G)** Depotentiation induced by LFS (2 Hz, 10 min) was reduced in p110γ mice (p <0.01, Student’s t-test).

p110γ or control red fluorescent protein, tdTomato, was delivered to the hippocampal CA1 region by AAV vector, driven by a CaMKIIα promoter for neuron-specific expression. Flag-tagged p110γ as well as the tdTomato were expressed in both the soma and the dendrites of the CA1 neurons (Figure 1A).

We first investigated the effects of p110γ overexpression on electrophysiological properties in the CA1 region of hippocampus. Analysis of basal synaptic transmission showed no significant difference in input/output relationships in PI3Kγ overexpressed slices compared to the control group (PI3Kγ overexpressed: n = 9, 6 animals; control: n = 11, 7 animals, Figure 1B, C). Paired-pulse facilitation (PPF) was also unaffected, indicating normal presynaptic release probability (PI3Kγ overexpressed: n = 9, 6 animals; control: n = 11, 7 animals, Figure 1D). Taken together, these results indicate that basal synaptic transmission was not changed by p110γ overexpression in the hippocampal Schaffer collateral (SC)-CA1 synapses.

We then probed the role of p110γ overexpression with regards to activity-dependent synaptic plasticity. The amount of LTP induced by a brief tetanus (100 Hz, 1 s) was not significantly different between the p110γ overexpression group and the control group (PI3Kγ overexpressed: n = 11, 127 ± 6% of baseline, 6 animals; control: n = 12, 123 ± 4% of baseline, 6 animals, Figure 1E). On the other hand, LTD was barely induced by repetitive low-frequency stimulations (LFS, 1 Hz, 15 min) in the p110γ overexpressed slices compared to the tdTomato expressed control group (Figure 1F). Field EPSP slope measured 1 hr after LTD induction for the p110γ and control group was 96 ± 5% (n = 10, 7 animals) and 77 ± 4% of baseline (n = 12, 8 animals), respectively (Student’s t-test, p <0.01). Moreover, we identified that the reversal of LTP (i.e., depotentiation) by a train of LFS (2 Hz, 10 min) was reduced in the p110γ overexpression group (n = 10, 7 animals, 127 ± 6 of pre-LTP baseline) in contrast to the control group (n = 12, 8 animals, 85 ± 11% of baseline), indicating synapses become less plastic following p110γ overexpression (Student’s t-test, p <0.01, Figure 1G).

### Molecular mechanisms of impaired NMDAR-LTD in p110γ-overexpressed hippocampal slices

**Figure 2:**
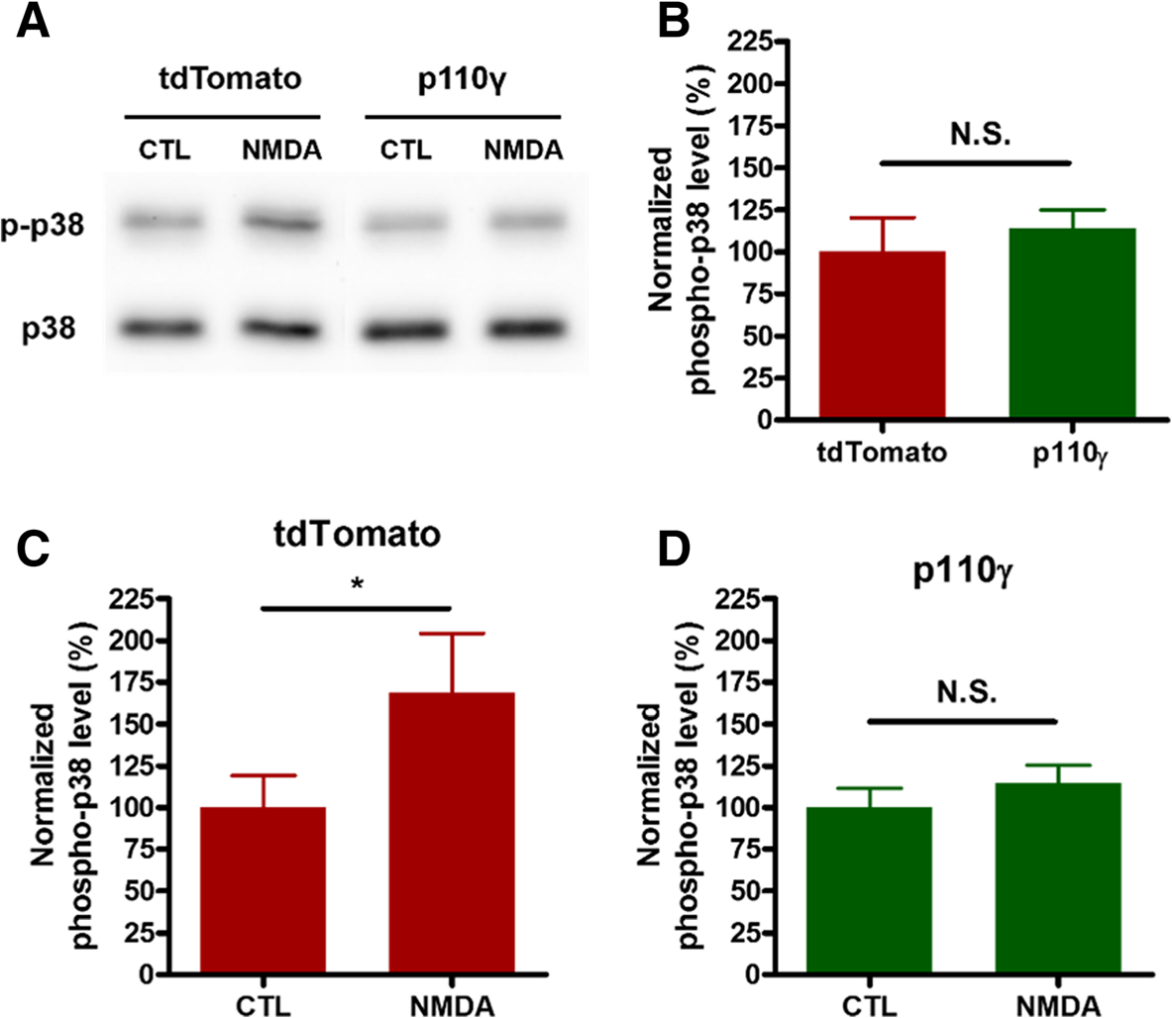
**Impaired induction of p38 MAPK phosphorylation by chemical LTD in p110γ-overexpressed hippocampal CA1 region. (A)** Representative phospho/total p38 MAPK western blot from tdTomato- or p110γ-overexpressed slices after chemical LTD induction (NMDA treatment). **(B)** Normalized basal phosphorylation level of p38 MAPK from tdTomato- or p110γ-overexpressed slices. **(C)** Normalized phosphorylation level of p38 MAPK from tdTomato-overexpressed slices after chemical LTD induction (n = 5, p <0.05, paired Student’s t-test). **(D)** Normalized phosphorylation level of p38 MAPK from p110γ-overexpressed slices, after chemical LTD induction (n = 5, NS, paired Student’s t-test).

We next examined the effect of p110γ overexpression on the downstream pathway involved in NMDAR-LTD. p38 MAPK is known to be involved in NMDAR-LTD through Rap1 signaling pathway and active Rap1 directly binds to p110γ [10-12]. In our previous study, we found that p110γ deletion reduced the phosphorylation of p38 MAPK induced by chemical LTD [9]. We performed similar chemical LTD experiments with tdTomato- or p110γ-overexpressing hippocampal slices. We first confirmed that p110γ overexpression itself did not affect the basal phosphorylation level of p38 MAPK (n = 5, Figure 2A, B). The phosphorylation of p38 MAPK (p-p38) was significantly increased in response to the chemical LTD induction in the tdTomato-expressing group (Figure 2A, C). However, the increase in p38 MAPK phosphorylation associated with chemical LTD was impaired in p110γ-overexpressing group (Figure 2A, D). These results support the finding that NMDAR-LTD is impaired by p110γ overexpression and suggest that this might be due to impairment in p38 MAPK signaling.

### Effect of p110γ overexpression in the hippocampal CA1 region on anxiety and locomotor activity

**Figure 3:**
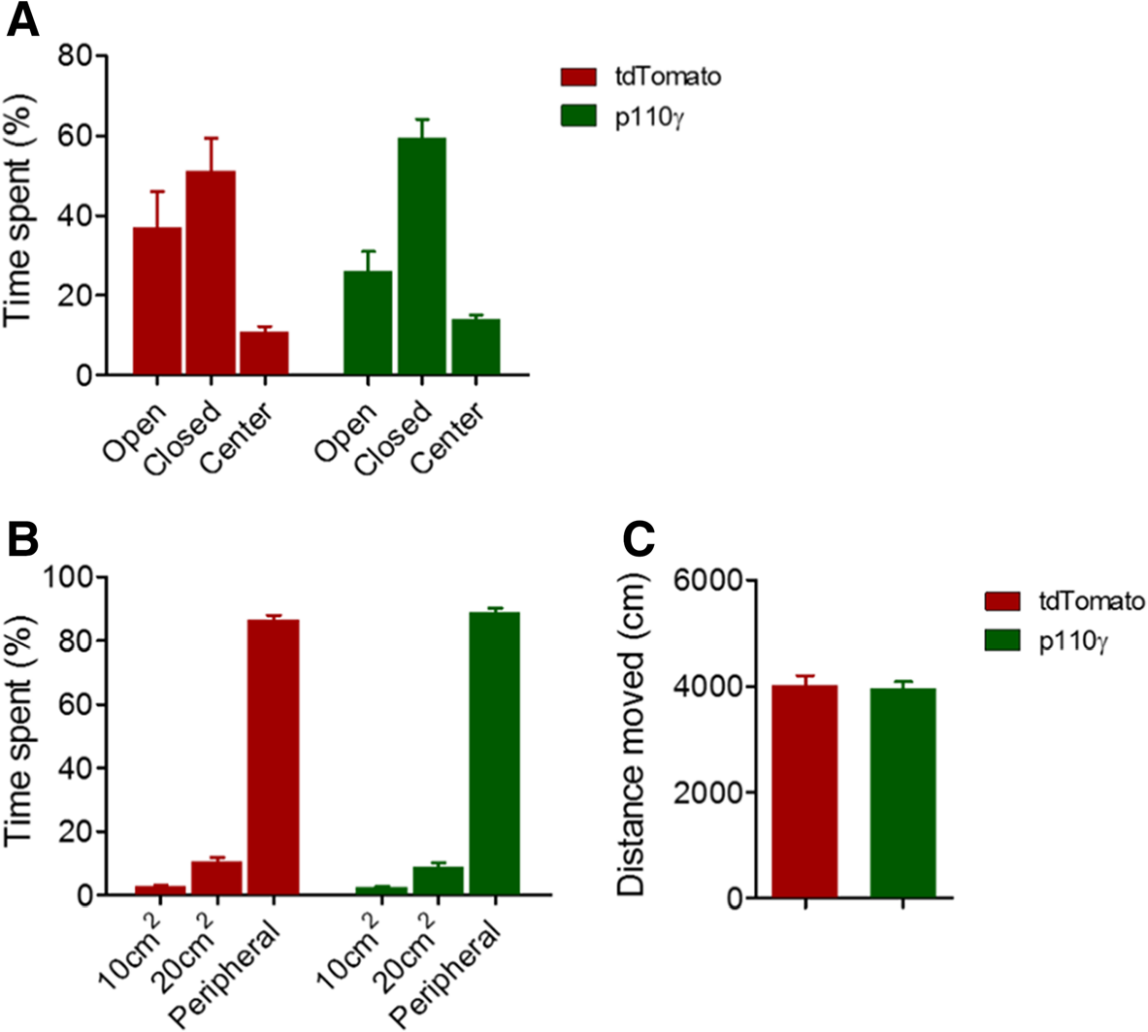
**Effect of p110γ overexpression on basal anxiety and locomotion. (A)** Basal anxiety levels of p110γ-or tdTomato-overexpressed mice in the elevated plus maze (EPM) test (Flag-p110γ, n = 12; tdTomato, n = 9, tested at 2 weeks after AAV injection). **(B)** Basal locomotive activity in the open field (OFT) test (Flag-p110γ, n = 14; tdTomato, n = 12, tested at 15 days after AAV injection). **(C)** Total distance moved (cm) during 100 s in the OFT.

Next, we examined the effect of p110γ overexpression on basal behavioral tasks. We performed a series of behavioral experiments where basal anxiety and locomotion were measured. In the elevated plus maze (EPM) test, which is a well-established method to assess anxiety level in rodents, the p110γ-overexpressed group exhibited a similar level of anxiety compared to the control group (there was a tendency to be more anxious in the p110γ-overexpressed group without any statistical significance; Figure 3A). In addition, the anxiety level and the locomotive activity of the p110γ–overexpressed group were similar to those of the control group in the open field test (OFT) (Figure 3B, C), indicating that the exogenous expression of p110γ does not affect basal rodent behaviors such as anxiety and locomotion.

### Effect of p110γ overexpression on hippocampus-dependent learning tasks

**Figure 4:**
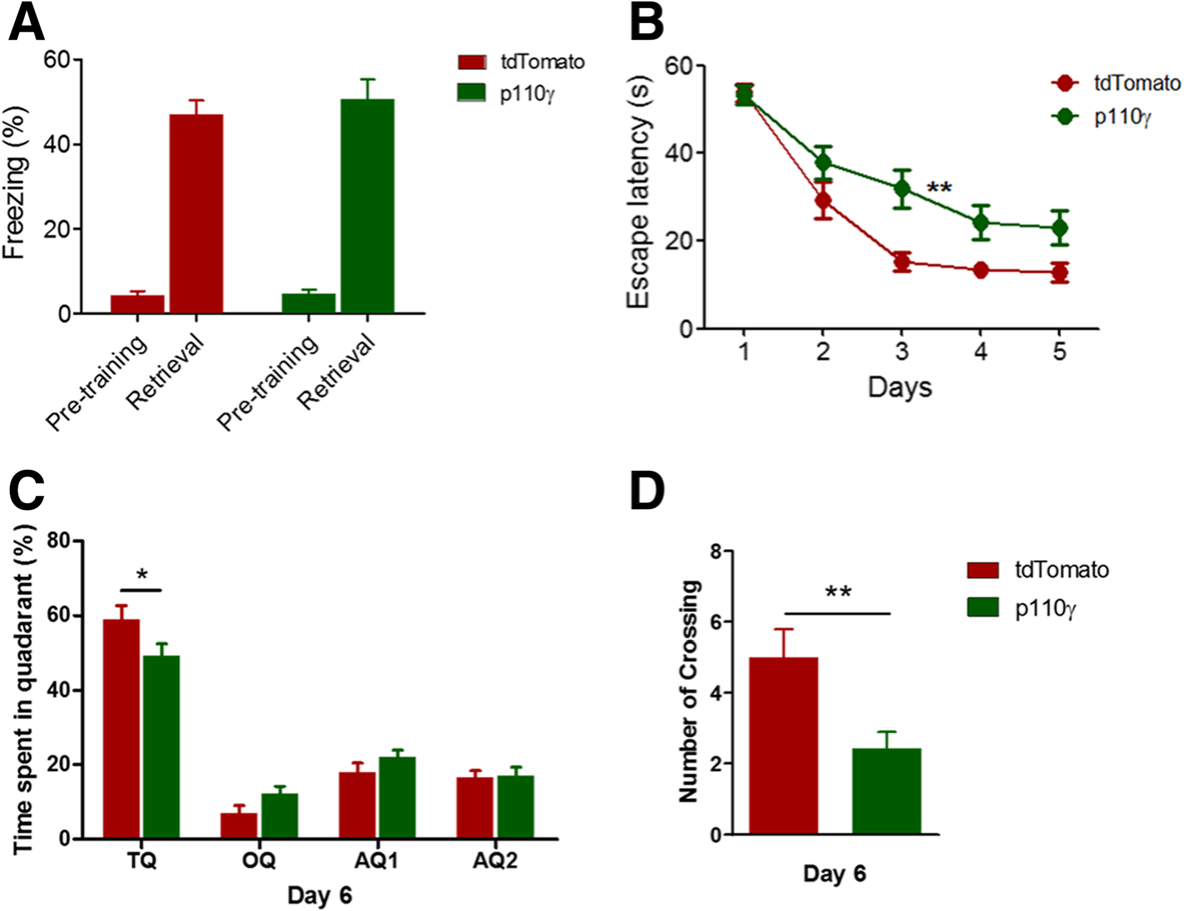
**Spatial learning in the Morris Water Maze (MWM) Task was reduced in p110γ-γ-γ-. (A)** Contextual fear memory of p110γ- or tdTomato-expressed mice (Flag-p110γ, n = 14; tdTomato, n = 12). **(B)** Average escape time traveled to the hidden platform (mean ± sem) in the MWM test (flag-p110γ, n = 14; tdTomato, n = 12, F = 8.25, **p <0.01, repeated measures two-way ANOVA). **(C)** The average percentage of time spent in the target quadrant (TQ, *p <0.05, repeated measures two-way ANOVA), the opposite quadrant (OQ) and two adjacent quadrants (AQ) during the probe trial given on day 6 of the MWM task. **(D)** Total number of crossings of the location of the platform (**p <0.01, Student’s t-test) during the probe trials.

We next investigated the effect of p110γ overexpression on hippocampus-dependent learning tasks. Our previous study showed that without pre-exposure to the context in contextual fear conditioning, the freezing level was reduced in *pi3kcg*^*−/−*^ mice [9]. Furthermore, in the reversal learning session of the Morris Water Maze (MWM) task, time spent in the old-target quadrant where the platform was located during the initial training sessions was increased in *pi3kcg*^*−/−*^ mice [9]. These results suggested that PI3Kγ plays a critical role in hippocampus-dependent learning tasks. Therefore, we wanted to determine whether contextual fear memory and spatial learning were affected by p110γ overexpression. We performed contextual fear conditioning and MWM test with the same protocols used in the previous study [9]. In the contextual fear memory test, the p110γ-overexpressed group showed equivalent freezing levels compared to the control group (Figure 4A), suggesting that p110γ overexpression in the hippocampus did not enhance or impair fear memory formation. However, spatial learning in the MWM test was significantly disrupted by overexpression of p110γ (Figure 4B). In addition, the p110γ overexpression group exhibited a tendency to spend less time in the target quadrant and fewer number of platform crossings during the probe test on day 6, suggesting that p110γ overexpression has a negative impact on spatial memory (Figure 4C, D).

## Discussion

We investigated the behavioral and electrophysiological effects of PI3Kγ overexpression in the hippocampal CA1 region of mice. Our previous experiments with Pik3cg^−/−^ mice demonstrated that PI3Kγ was critically involved in NMDA receptor-dependent LTD (NMDAR-LTD) and reversal learning in the MWM [9]. To further elucidate the role of PI3Kγ in synaptic plasticity and learning, we used an AAV-mediated genetic delivery of p110γ, a catalytic subunit of PI3Kγ, to the hippocampal CA1 neurons.

We found that the overexpression of p110γ in the CA1 region disrupted NMDAR-LTD and depotentiation of potentiated synapses and that this effect was associated with a reduced phosphorylation level of p38 MAPK in response to LTD induction. In contrast, LTP in the p110γ overexpression group was normal. Hippocampus-dependent spatial memory in the Morris water maze (MWM) task was also impaired. The finding that a similar impairment in NMDAR-LTD results from either p110γ knockout or overexpression indicates that an appropriate level of p110γ is required for normal LTD. This idea is further supported by the similar effects on p38 MAPK phosphorylation by the knockout and overexpression of p110γ. It is possible that p110γ is necessary for p38 MAPK activation, while overexpressing p110γ results in saturation or compensation of the pathway leading to similar impairment of p38 MAPK activation. Overexpression of p110γ additionally impaired depotentiation which also involves p38 MAPK signaling [13]. However, since a similar impairment of p38 MAPK activation in Pik3cg^−/−^ mice was not associated with disruption of depotentiation, the impaired depotentiation in p110γ overexpressing slices is likely due to disruption of an additional signaling pathway specifically involved in depotentiation. Further studies are required to establish the underlying mechanisms.

A significant amount of evidence has indicated that hippocampal LTD contributes to learning and memory [14-19]. Various genetic and pharmacological studies have shown that animals having impaired LTD show various behavioral phenotypes, some showing similar impairment in water maze task as the present study [16,18]. There seems to be differential role of each pathways required for LTD in various behaviors, and these behavioral phenotypes cannot be simply classified as a general result of LTD impairment. Both p110γ knockout and overexpression resulted in LTD impairment while these two opposite manipulations resulted in different behavioral phenotypes. Spatial learning during initial training in Pik3cg^−/−^ mice was intact but reversal learning was impaired [9]. In contrast, p110γ-overexpressed mice showed impairment in spatial learning during initial training. These results indicate that p110γ is multi-functional, required for spatial learning and reversal of the initial spatial memory in different ways. The role of hippocampal depotentiation in learning and memory is less clearly established. Therefore, we cannot exclude the possibility that impaired depotentiation might be involved in the spatial learning deficit in the p110γ-overexpressing mice.

In conclusion, our present findings have added further evidence to support a role for PI3Kγ in both NMDAR-LTD and hippocampus-dependent learning and memory.
